# Evaluating trajectories of episodic memory in normal cognition and mild cognitive impairment: Results from ADNI

**DOI:** 10.1371/journal.pone.0212435

**Published:** 2019-02-25

**Authors:** Xiuhua Ding, Richard J. Charnigo, Frederick A. Schmitt, Richard J. Kryscio, Erin L. Abner, for the Alzheimer’s Disease Neuroimaging Initiative

**Affiliations:** 1 Western Kentucky University, Department of Public Health, Bowling Green, Kentucky, United States of America; 2 University of Kentucky, Department of Statistics, Lexington, Kentucky, United States of America; 3 University of Kentucky, Department of Biostatistics, Lexington, Kentucky, United States of America; 4 University of Kentucky, Sanders-Brown Center on Aging, Lexington, Kentucky, United States of America; 5 University of Kentucky, Department of Neurology, Lexington, Kentucky, United States of America; 6 University of Kentucky, Department of Epidemiology, Lexington, Kentucky, United States of America; Nathan S Kline Institute, UNITED STATES

## Abstract

**Background:**

Memory assessment is a key factor for the diagnosis of cognitive impairment. However, memory performance over time may be quite heterogeneous within diagnostic groups.

**Method:**

To identify latent trajectories in memory performance and their associated risk factors, we analyzed data from Alzheimer’s Disease Neuroimaging Initiative (ADNI) participants who were classified either as cognitively normal or as Mild Cognitive Impairment (MCI) at baseline and were administered the Rey Auditory Verbal Learning test (RAVLT) for up to 9 years. Group-based trajectory modeling on the 30-minute RAVLT delayed recall score was applied separately to the two baseline diagnostic groups.

**Results:**

There were 219 normal subjects with mean age 75.9 (range from 59.9 to 89.6) and 52.5% male participants, and 372 MCI subjects with mean age 74.8 (range from 55.1 to 89.3) and 63.7% male participants included in the analysis. For normal subjects, six trajectories were identified. Trajectories were classified into three types, determined by the shape, each of which may comprise more than one trajectory: stable (~30% of subjects), curvilinear decline (~ 28%), and linear decline (~ 42%). Notably, none of the normal subjects assigned to the stable stratum progressed to dementia during the study period. In contrast, all trajectories identified for the MCI group tended to decline, although some participants were later re-diagnosed with normal cognition. Age, sex, and education were significantly associated with trajectory membership for both diagnostic groups, while *APOE* ɛ4 was only significantly associated with trajectories among MCI participants.

**Conclusion:**

Memory trajectory is a strong indicator of dementia risk. If likely trajectory of memory performance can be identified early, such work may allow clinicians to monitor or predict progression of individual patient cognition. This work also shows the importance of longitudinal cognitive testing and monitoring.

## Introduction

From a clinical and research perspective, an individual’s cognition may be categorized as unimpaired (normal cognition), mildly impaired (mild cognitive impairment or MCI), or moderately to severely impaired (dementia). Over time, those with normal cognition remain stable or decline to MCI or dementia. Similarly, those with MCI may remain stable (about 61% based on Wolf’s study), progress to dementia (annual progression rate is about 9.6% in specialist settings and about 4.9% conducted in community settings), or revert to normal (about 19.5%)[1–4]. Therefore, examining potential trajectories within populations and identifying individuals who are likely to follow particular cognitive trajectories could inform early diagnosis and predict progression. However, most analyses of cognitive test data, such as from linear mixed models, only provide a mean score to describe the average change for the study population over the follow-up time [5–7]. The different underlying developmental courses within certain diagnostic groups are poorly understood due to the limitations of the statistical methods. Group-Based Trajectory Modeling (GBTM), developed by Nagin [8, 9], provides a solution for this issue. It assumes that the underlying population (such as people with MCI) is a mixture of at least two latent subgroups. Individuals in each latent subgroup follow a similar trajectory over time. Results of this analysis provide estimated longitudinal trajectories, sometimes called “developmental trajectories”, and the procedure provides the estimated proportion of each sub-group following the same latent trajectory.

Memory assessment in neuropsychological testing is one of key elements in the diagnosis of MCI and dementia [10]. One of most commonly used tests for verbal memory assessment is the Rey Auditory Verbal Learning Test (RAVLT) [11], which is designed to evaluate episodic memory in persons age 16 and older [12]. The RAVLT provides measures of immediate memory span, learning, and delayed recall, so the severity of memory dysfunction and changes over time can be evaluated. For instance, MCI patients show poorer learning than ‘recovered’ MCI and healthy control groups [13]. The RAVLT is easily administered, so researchers often prefer it to other list learning tests, especially under conditions of limited assessment time [14]. RAVLT performance is influenced by subjects’ demographic characteristics, including age, education, and sex [15]. The RAVLT delayed recall score has been reported to have adequate discrimination in older adults with normal cognition vs. MCI (AUC = 0.71) and good discrimination for normal cognition vs. dementia (AUC = 0.93) [16].

Poor performance on the test is considered a prognostic marker for MCI and dementia [17]. Zhao et al.’s [18] study shows that RAVLT performs better than the Complex Figure Test (CFT) for predicting progression from MCI to AD, and data from the Canadian Study of Health and Aging demonstrate that RAVLT short delayed recall may be used to predict incident dementia (sensitivity = 78%, specificity = 72%, positive likelihood ratio = 2.81 when combined with Wechsler Adult Intelligence Test Revised (WAISR) Digit Symbol).[19]. In the Gothenburg MCI study [20], neuropsychological tests including RAVLT, along with hippocampal volume and cerebrospinal fluid markers, were used to predict progression from MCI to dementia within a follow-up time of two years. They found that a combination of all markers was the most successful in predicting dementia, but the RAVLT was the best individual predictor (AUC = 0.93) for dementia. RAVLT was also used to distinguish the AD from other types of dementia [21].

In this analysis, we explored latent trajectories of episodic memory using GBTM and longitudinal RAVLT measures within two diagnostic groups: Alzheimer’s Disease Neuroimaging Initiative Phase 1 (ADNI1) subjects with a diagnosis of normal cognition or MCI at study baseline. Also, we investigated whether trajectory membership predicted incident dementia.

## Materials and methods

### Sample and data sources

Data were obtained and downloaded from the ADNI database (http://adni.loni.usc.edu/) on June 3, 2015. The primary goal of ADNI project is to obtain and assess clinical, imaging, genetic and biospecimen biomarkers related to the development and progression of the AD and develop treatments that may slow the progression of AD [22]. More information can be found at www.adni-info.org.

Because our interest is focused on longitudinal change, our analysis was limited to ADNI1 participants, who have the longest follow-up. During ADNI1, which began recruiting participants in 2004, 400 MCI participants, 200 participants with early AD, and 200 control participants, all aged 55–90 years, were targeted for recruitment at 50 study sites across North America (actual enrollment: 397 MCI participants, 189 early AD, and 229 normal control participants). They were followed-up at regular intervals from study baseline. Baseline MCI subjects were followed-up every six months for the first three years and then yearly after that. Baseline normal subjects were followed-up every six months for the first year and then yearly after that. ADNI1 has the following inclusion criteria for all subjects: 1) Hachinski Ischemic Score less than or equal to 4; 2) Age between 55–90; 3) Geriatric Depression Scale less than 6; 4) Visual and auditory acuity adequate for neuropsychological testing; 5) Good general health with no diseases precluding enrollment; 6) At least a 6^th^ grade education. Participants were classified as normal cognition or MCI based on criteria in Table 1 (more details can be found in ADNI website: http://adni.loni.usc.edu/).

**Table 1:** ADNI1 diagnostic criteria for normal cognition and MCI.

	Normal	MCI
Memory complaint	No	Yes
Logical Memory II subscale (Delayed Paragraph Recall)	a) ≤ 9 for 16 or more years of education b) ≤ 5 for 8–15 more years of education c) ≤ 3 for 0–7 years of education	a) ≤ 8 for 16 or more years of education b) ≤ 4 for 8–15 more years of education c) ≤ 2 for 0–7 years of education
Mini-Mental State Exam score	24–30 (inclusive)	24–30 (inclusive)
Clinical Dementia Rating	0	0.5
Memory Box score	0	At least 0.5
Cognition	Absence of significant impairment in cognitive functions or activities of daily living.	General cognition and functional performance sufficiently preserved such that a diagnosis of Alzheimer’s disease cannot be made by the site physician at the time of the screening visit.

All ADNI research activities were approved by Institutional Review Boards (IRB) at the participating study sites, and all participants provided written informed consent. The University of Kentucky IRB declared this secondary analysis of ADNI data exempt since the ADNI data are de-identified.

### Inclusion and exclusion criteria

All analyses for the current study were based on participants diagnosed with MCI or normal cognition who enrolled in ADNI1 and had any follow-up visits in ADNI1, ADNIGO, or ADNI2. Fourteen participants (1 American Indian, 12 Asian, and one more than one race) were excluded because their numbers were too small for further analysis. Twenty-one participants with only one visit were also removed from the analyses, which resulted in 591 total participants for analysis: 219 normal participants and 372 MCI participants. No statistical significances were found among baseline age, sex, education, and baseline MMSE total scores between included and excluded participants.

### Rey auditory verbal learning test (RAVLT)

The RAVLT is a list-learning task that measures auditory verbal memory [23]. The RAVLT is conducted using two 15-item lists of unrelated words (List A and List B) that are read to the participant in a series of trials. To begin, List A is read to the participant, and the participant is asked to repeat as many of the 15 words as they can, and the number of correct words is recorded. This procedure is repeated in another four trials, which results in 5 learning trial scores. Then the examiner reads the second list of 15 words (List B) to the participant, and the participant is asked to recall as many of words in List B as possible. Next, the participant is again asked to recall the words in List A, and the number of words (immediate recall score) correctly recalled is recorded. The participant is then given different tasks to do for 30 minutes. After 30 minutes, the participant is asked again to recall as many words as they can from List A, and the number of correct words (30-minute delayed recall) is recorded. Last, the participant is asked to recognize the words in List A when presented a sheet containing the 15 List A words plus 15 distractor words, and examiner records the number of successes (recognition score). In the current study, the 30-mintute delayed recall score, which ranges from 0 to 15 [24], is the outcome of interest.

### Covariates

Covariates included *APOE* genotype, baseline age, race, sex, years of education, smoking information, and body mass index (BMI), as well as self-reported indicators of cardiovascular disease risk (i.e., diabetes, and hypertension) and sleep apnea. *APOE* genotype, which has been shown to be associated with cognitive trajectory [25], was available for all 591 participants (ɛ2/2: 2 (0.34%); ɛ2/3: 46 (7.78%); ɛ2/4: 13 (2.20%); ɛ3/3: 281 (47.55%); ɛ3/4: 199 (33.67%); ɛ4/4: 50(8.46%)). The genotypes were converted to a dummy indicator for a carrier of at least one ɛ4 allele. Age at baseline was calculated based on the participant’s birthdate and visit date. Race was coded as a dummy variable: 0 (Black) and 1 (White). Similarly, smoking was coded as 0 (non-smoker) and 1 (current smoker). Since ADNI collects medical history as single-field text strings (variable “mhdesc”), the self-reported status of hypertension, diabetes, and sleep apnea was extracted by searching for keywords. For example, participants with sleep apnea were identified by first converting all “mhdesc” text string values to uppercase, and then a search for the text string ‘SLEEP’ was used to find participants who reported sleep problems. Then each identified case was checked individually to confirm sleep apnea. A similar procedure was conducted for the status of hypertension (keywords: “HYPERTENSION,” “HIGH BLOOD PRESSURE”) and diabetes (keyword: DIABETES). Misspelled conditions in the raw data were identified when each value was checked. These three variables were coded as dummy variables (0 = not reported and 1 = reported).

### Statistical analysis

Analyses were conducted in this study as follows. First, analyses on baseline differences of characteristics between normal and MCI participants were assessed with Chi-square, t test, or the Mann-Whitney test. Second, GBTM was applied to identify latent longitudinal trajectories of RAVLT 30-minute delayed recall scores for normal and MCI participants separately. For implementing GBTM, the mean level of the outcome was modeled first as a function of time, and latent groups were identified; the proportion of the population that follows each latent trajectory was estimated based on posterior probablities of group memberships. Individuals were assigned to specific latent groups based on the maximum posterior probability of group membership for each. Next, we compared individuals’ cognitive status at enrollment and cognitive status at the end of follow-up by each trajectory. Finally, we also examined how the probability of trajectory group membership varied with covariates versus an arbitrary reference trajectory group.

**Fig 1:**
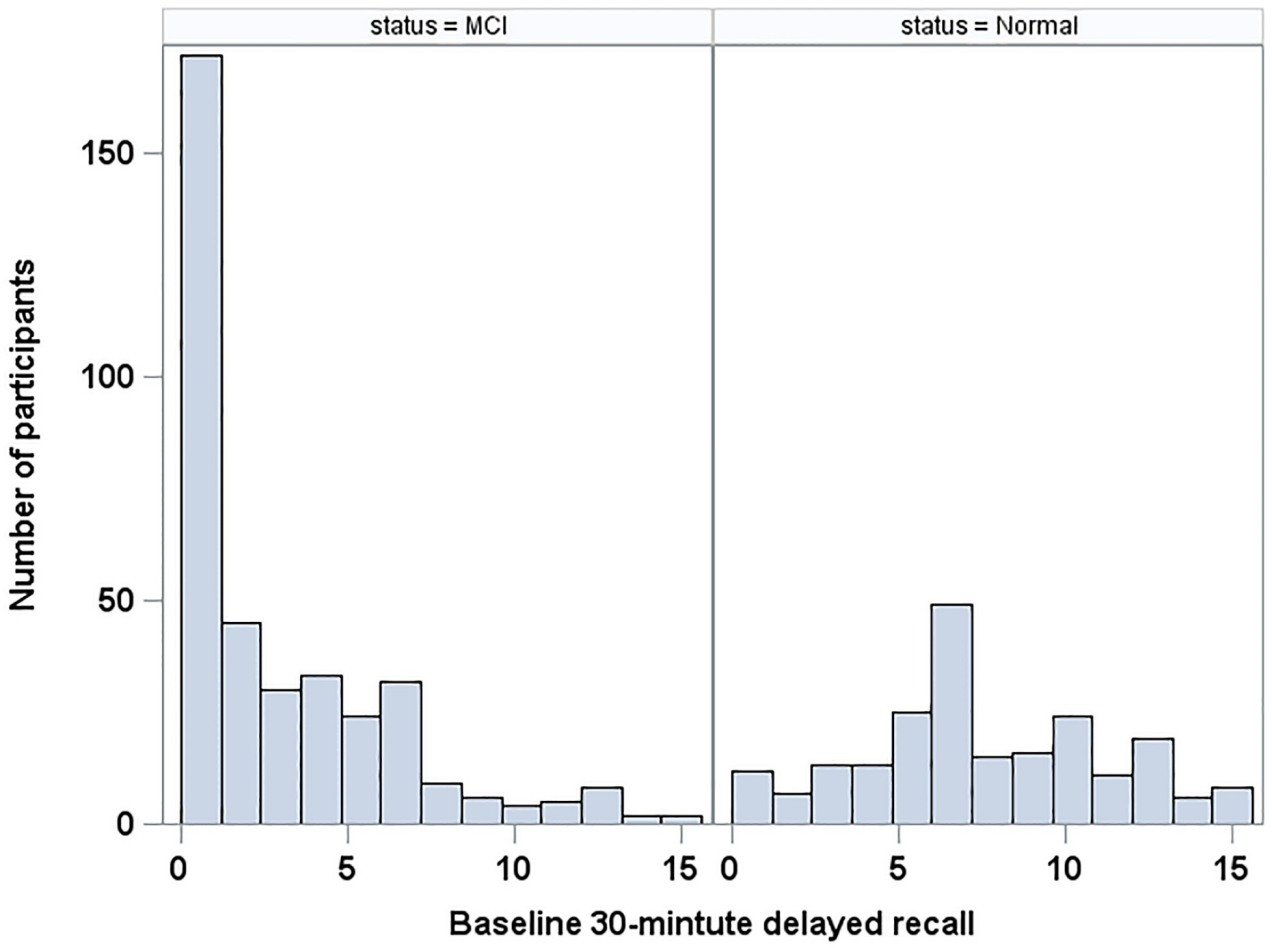
Frequency plot of baseline RAVLT 30-minute delayed recall scores for MCI and normal participants.

**Fig 2:**
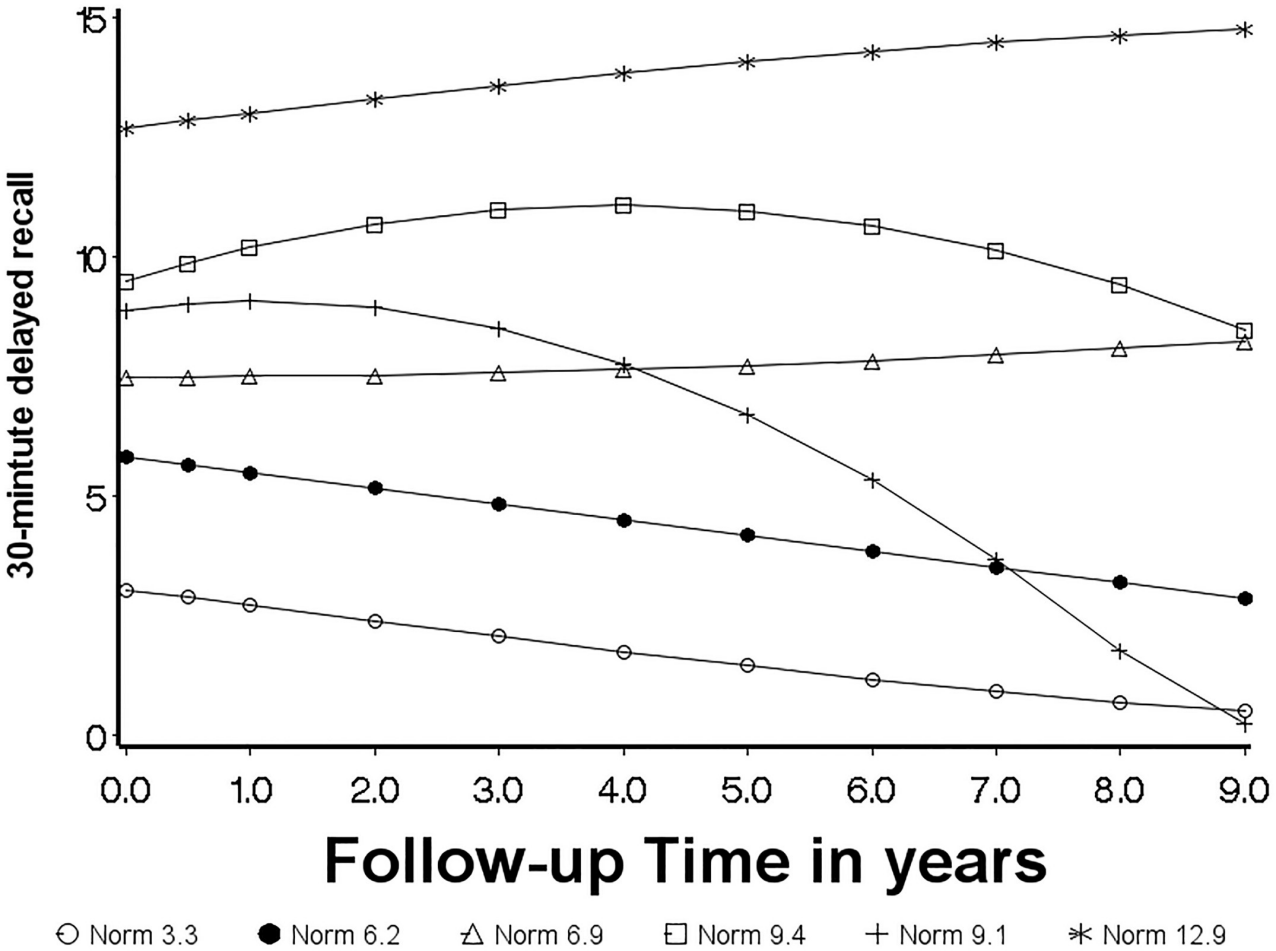
Model based trajectories identified for baseline normal ADNI participants.

**Fig 3:**
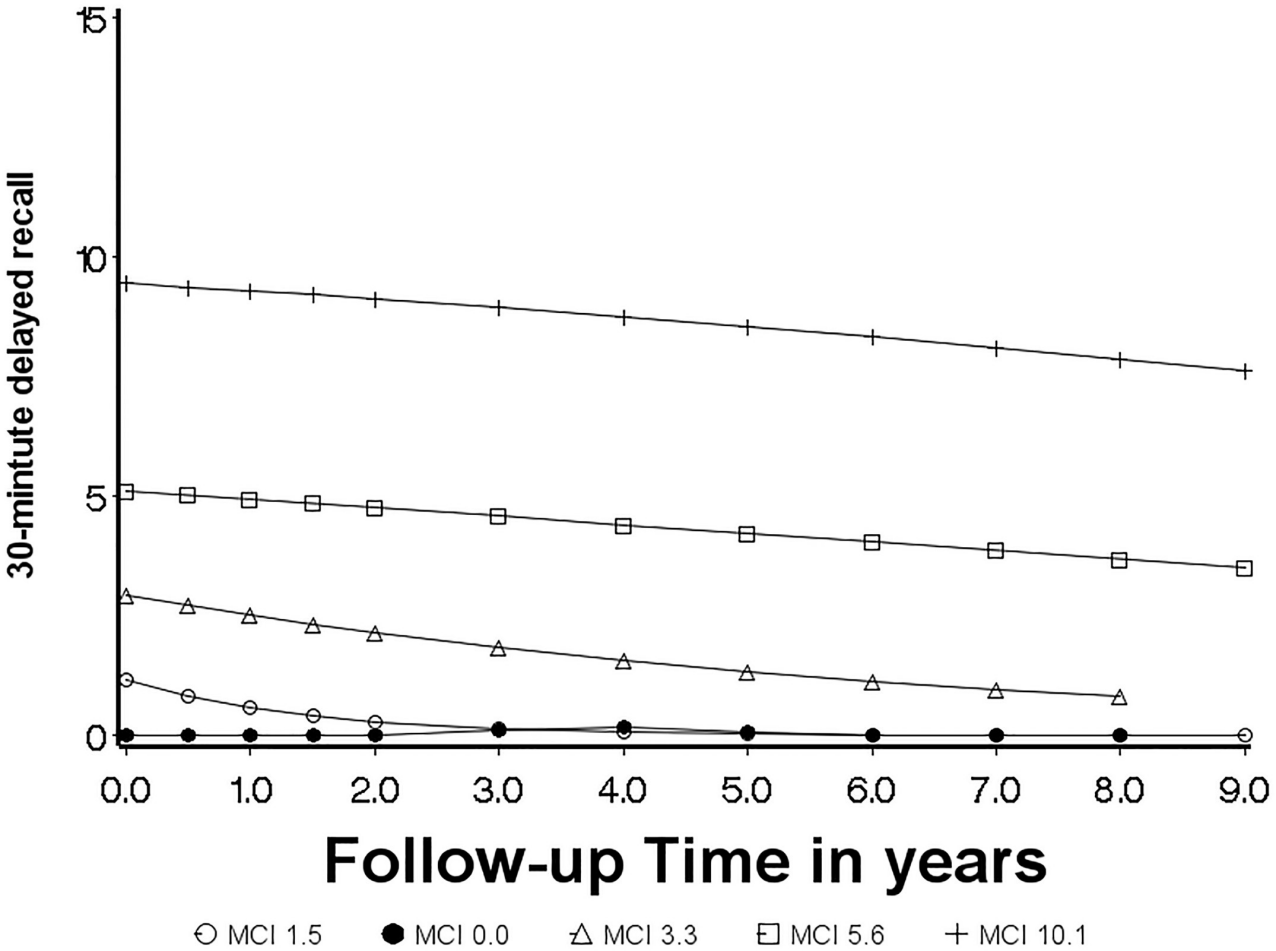
Model based trajectories identified for baseline MCI ADNI participants.

To identify the best fitting GBTM models, various models were fitted for 2 to 6 trajectories (inclusive) [6] and all combinations of orders (quadratic was the highest order) of each group. All covariates were included and fixed at baseline. Bayesian Information Criterion (BIC) was applied to select the optimal number of groups and orders [8]. Then, log-likelihood ratio tests were applied to reduce the number of covariates in the model. GBTM accommodates different types of outcome data including count, psychometric scale, and dichotomous data [9]. Based on histograms of the outcome in each subsample (see Fig 1), we assumed that the 30-minute delayed recall scores followed a censored (i.e., bounded) normal distribution for the normal group and a zero-inflated Poisson (ZIP) distribution for the MCI group, which showed evidence of excess zero scores. In the normal group, the 30-minute delayed recall score was standardized by subtracting the baseline sample mean (7.5) and dividing by the sample standard deviation (3). The estimated scores were transformed back to the original scale when the figures (Figs 2 and 3) were plotted. For simplicity in the ZIP model for the MCI group, we assumed that the probability for the excess zero generating process was common to all trajectory groups and constant over time.

The final fitted models provide descriptive information on the estimated groups, which include: (1) posterior probabilities of an individual belonging to one of the identified groups, (2) the proportion of each potential trajectory group following the same latent trajectory, (3) regression parameters to define the shape of the trajectories over time (intercept only, linear, and quadratic in the present study), and (4) risk and protective factors associated with membership in a trajectory group. All data were analyzed using PC-SAS 9.4 (SAS Institute, Inc., Cary, NC), and 0.05 was set as the significance level. Group trajectory analyses were carried out using the procedure PROC TRAJ [5].

## Results

### General characteristics of participants in the study sample

Table 2 presents the characteristics of participants overall and by cognitive status at baseline. Baseline normal participants (n = 219) were followed up longer than MCI participants (n = 372) (p < 0.001). Normal participants were older (p = 0.039), more highly educated (p = 0.049), more likely to be female (p = 0.007), and had higher BMI than MCI participants (p = 0.037). Normal participants comprised fewer *APOE-ɛ4* allele carriers and participants with sleep apnea than the MCI group (p<0.001). Over 91% of participants had at least three examinations. There were 2476 total observations from MCI participants and 1541 observations from normal participants.

**Table 2:** Characteristics of ADNI1 participants by cognitive status.

Characteristic^a^^,^^b^^,^^c^^,^^e^	All (N = 591)	Normal (n = 219)	MCI (n = 372)	P value^d^
Number of examinations				
1/2/3/4+	0/27/25/539	0/7/4/208	0/20/21/331	
Median(interquartile range)	7(4)	9(5)	6(4)	<0.001
Months of follow-up				
Median(interquartile range)	48(48)	72(60)	36(48)	<0.001
Baseline age, y	75.2±6.6	75.9±5.1	74.8±7.3	0.039
Education, y	15.8±2.9	16.1±2.8	15.6±3.0	0.049
White race	562 (95.1)	204 (93.2)	358 (96.2)	0.094
Male sex	352 (59.6)	115 (52.5)	237 (63.7)	0.007
Baseline smoking	235 (39.8)	84 (38.4)	151 (40.6)	0.592
APOE-ɛ4 (≥1 ɛ4 allele)	262 (44.3)	58 (26.5)	204 (54.8)	<0.001
Baseline sleep apnea	60 (10.2)	14 (6.4)	46 (12.4)	0.020
Baseline hypertension	278 (47.1)	105 (48.0)	173 (46.6)	0.757
Baseline diabetes	49 (8.3)	19 (8.7)	30 (8.1)	0.802
Baseline BMI, kg/m^2^	26.4±4.1	26.8±4.3	26.1±4.0	0.037
Baseline MMSE	27.4±2.6	29.0±1.1	26.5±2.8	<0.001

Note:

^a^BMI: Body Mass Index;

^b^ mean ± standard deviation;

^c^count (%).

^d^ P values for continuous variables from t test statistics and P values for categorical variables from Chi-square test except that p values for number of examinations and months of follow-up were from Mann-Whitney-Wilcoxon;

^e^ Mini–Mental State Examination total score.

### Potential groups identified from cognitive normal and MCI by GBTM, respectively

GBTM was applied to test how many distinct trajectory groups fit normal and MCI sample in this study, separately. Six latent profiles were identified for normal subjects (Fig 2), while five profiles were identified for MCI participants (Fig 3) based on the BIC values among the candidate trajectory models. Table 3 shows detailed descriptions of the trajectories for normal and MCI participants, including the shape of each group trajectory and the number of probable members, parameter estimates of trajectories, and mean and standard deviation of posterior probabilities. As shown in Table 3, for all six normal groups and all 5 MCI groups, the averages of the posterior membership probabilities were greater than 0.7, which indicates that the models are acceptable based on the Nagin’s ‘rule of thumb’ on minimum average posterior probability [26].

**Table 3:** Description of estimated trajectories for baseline normal and MCI participants. Trajectory groups are named based on the baseline diagnosis and baseline mean RAVLT delayed recall.

Trajectory group	Trajectory Polynomial^a^	P value ^b^	Parameter estimates of trajectory group^c^			Posterior probability^d^ Mean±SD (min)
			Intercept (SE)	Slope (SE)	Quadratic (SE)	
*Baseline Normal participants*						
Norm 3.3	Linear	<0.001	-1.48 (0.05)	-0.11 (0.01)	-	94.9±11.6 (62)
Norm 6.2	Linear	<0.001	-0.56 (0.04)	-0.11 (0.01)	-	95.9±9.7 (51)
Norm 6.9	Quadratic	0.51	-0.0001 (0.01)	0.001 (0.03)	0.003 (0.004)	92.6±13.2 (56)
Norm 9.4	Quadratic	<0.001	0.67 (0.07)	0.27 (0.04)	-0.03 (0.006)	91.7±14.7 (54)
Norm 9.1	Quadratic	<0.001	0.47 (0.07)	0.11 (0.06)	-0.05 (0.006)	92.8±12.3 (52)
Norm 12.9	Linear	<0.001	1.74 (0.06)	0.11 (0.02)		97.0±10.3 (54)
*Baseline MCI participants*						
MCI 0.0	Quadratic	0.012	-9.94 (2.63)	4.20 (1.47)	-0.53 (0.21)	83.5±9.5 (61)
MCI 1.5	Linear	<0.001	0.25 (0.16)	-0.70 (0.08)	-	95.5±9.3 (53)
MCI 3.3	Linear	<0.001	1.18 (0.08)	-0.14 (0.03)	-	87.3±15.2 (38)
MCI 5.6	Linear	0.02	1.73 (0.05)	-0.02 (0.01)	-	90.5±13.5 (51)
MCI 10.1	Linear	0.61	2.34 (0.04)	-0.01 (0.01)	-	95.8±10.6 (52)

Note:

^a^ = highest term of polynomial for the trajectory group;

^b^ = p value for highest term of polynomial for the trajectory group;

^c^ = parameter estimates in each trajectory group (intercept, slope, quadratic), SE = standard error of each parameter estimate;

^d^ = average and standard deviation of greatest posterior probability for all participants.

To facilitate discussion, trajectories were labeled based on baseline cognitive diagnosis and the baseline mean score of 30-minute delayed (Figs 2 and 3, and Table 3). For example, ‘Norm 6.9’ means trajectory for normal subjects with average 30-mintute delayed recall = 6.9 at baseline. We further describe the six trajectories for normal subjects as three types of trajectories over time based on the progress over time: stable (Norm 12.9 and Norm 6.9), curvilinear decline (Norm 9.4 and Norm 9.1), and linear decline (Norm 3.3 and Norm 6.2) (Fig 2). The Norm 12.9 (n = 22) and Norm 6.9 (n = 44) groups remained relatively stable over nine years of follow-up, which account for about 30% of normal participants in the sample. Both Norm 9.4 and Norm 9.1 present curvilinear change indicated by the quadratic term in the model (Table 3) over time, but with different decline rate at the different time. Norm 9.1 (n = 30) showed a slow curvilinear decline during the first four years of follow-up and faster decline after four years, and Norm 9.4 (n = 31) revealed mild curvilinear decline (Fig 2). The individuals in Norm 3.3 and Norm 6.2 demonstrate linear decline overtime (Fig 2). Notably, some of the groups are differentiated primarily by their intercepts, such as groups of Norm 12.9, Norm 6.9, Norm 6.2, and Norm 3.3, which might suggest participants assigned in those groups with low baseline means were misclassified at the time they enrolled into ADNI (e.g., Norm 3.3 and Norm 6.2). In contrast to groups identified for normal participants, all potential trajectory groups for MCI participants showed the tendency to decline, except MCI 0.0, which starts near and stays around “0” (floor effect) (Fig 3).

### Comparison between cognitive status at enrollment and cognitive status at the end of follow-up for individuals in each trajectory

Since the baseline cognitive status was evaluated in ADNI1 without regards of RAVLT assessments, we were able to investigate whether the development of each trajectory predicts dementia by calculating the proportion of participants’ cognitive status at the end of follow-up by each trajectory (Table 4) within baseline normal and MCI participants, respectively. The majority of normal participants assigned to Norm 6.9 and Norm 12.9, who should be stable as shown in the trajectory, remained cognitively normal over nine years follow-up and only 5 (out of 66) participants in Norm 6.9 progressed to MCI status (Table 4). No participants in Norm 6.9 or Norm 12.9 progressed to dementia by the end of follow-up. Members of Norm 3.3, and Norm 9.1 were most likely to develop dementia by the end of follow-up (18% and 17% of group members, respectively), which demonstrates that baseline scores alone often poorly predict future cognitive status.

**Table 4:** Proportion/Number of participants in each trajectory group and cognitive status at end of follow-up.

Trajectory GroupTrajectory	%^a^	n^b^	Cognitive Diagnosis at end of follow-up^c^		
			Normal	MCI	Dementia
*Baseline normal*					
Norm 3.3	15.1	33	19 (57.6)	8 (24.2)	6 (18.2)
Norm 6.2	26.6	58	39 (67.2)	14 (24.1)	5 (8.6)
Norm 6.9	20.2	44	39 (88.6)	5 (11.4)	0 (0.0)
Norm 9.4	14.2	31	28 (90.3)	2 (6.5)	1 (3.2)
Norm 9.1	13.8	30	20 (66.7)	5 (16.7)	5 (16.7)
Norm 12.9	10.1	22	22 (100.0)	0 (0.0)	0 (0.0)
*Baseline MCI*					
MCI 1.5	38.5	143	0 (0.0)	39 (27.3)	104 (72.7)
MCI 0.0	17.8	66	0 (0.0)	19 (28.8)	47 (71.2)
MCI 3.3	17.8	66	0 (0.0)	34 (51.5)	32 (48.5)
MCI 5.6	19.7	73	8 (11.0)	51 (69.9)	14 (19.2)
MCI 10.1	6.2	23	5 (21.7)	15 (65.2)	3 (13.0)

Note:

%^a^ = percent of subjects in trajectory group with each diagnosis based on the greatest posterior probability for the subject;

n^b^ = number of subjects assigned in the trajectory group;

^c^ = count (%).

Similarly, for baseline MCI, participants in MCI 1.5 (n = 143) and MCI 0.0 (n = 66) were most likely to develop dementia by the end of follow-up, with over 70% of each group progressing (Table 4). Participants in MCI 3.3 (n = 66) had a slightly better chance to remain in MCI (52%) than develop dementia (48%), while the majority of participants in MCI 5.6 (70%) and MCI 10.1 (65%) remained MCI. Interestingly, 11% of participants in MCI 5.6 (n = 73), and 22% of participants in MCI 10.1 (n = 23) were re-diagnosed with normal cognition by the end of follow-up. Again, baseline performance was not a good predictor of future cognitive status since participants’ cognitive status can convert, be stable, or progress to dementia. No participants in the groups of MCI 1.5, MCI 0.0, and MCI 3.3 were diagnosed as normal by the end of follow-up.

### Risk factors associated with the probability of trajectory group membership

Analyses also were done to examine the factors may influence group membership. Tables 5 and 6 present the parameter estimates for the risk factors associated with trajectory group membership in normal and MCI participants, respectively. The comparison groups were arbitrarily selected for both normal (Norm 9.4) and MCI (MCI 5.6) participants. Based on BIC and log-likelihood ratio test, age, BMI, and education were retained in both the 6-group model for normal participants and 5-group MCI model (Tables 5 and 6), while sex was only retained in the model for normal participants, and *APOE* ɛ4 was in the model only for MCI participants. Demographic variables associated with group memberships among baseline normal participants (vs. Norm 9.4) included female sex (p = 0.02 for Norm 12.9), older age (p = 0.03 for Norm 6.2) and higher education (p = 0.02 for Norm 6.2, and p = 0.01 for Norm 6.9). For example, in Norm 6.2 group for normal baseline participants, it was estimated that each additional year of education increase reduces ratio of the probability of belonging to Norm 6.2 vs. the probability of belonging to Norm 9.4 by 22%. Similar effects were observed for Norm 3.3 vs. Norm 9.4. Presence of *APOE* ɛ4 allele increased the probability ratio of belonging to MCI 1.5 vs. MCI 5.6 by 85%, and the probability ratio of belonging to MCI 0.0 vs. MCI 5.6 by 388%, holding other covariates in the model constant. Based on Table 6 for MCI participants, age is not significant but kept in the model, which may suggest that age cannot distinguish the rest groups from reference group MCI 5.6, but it may distinguish MCI 3.3 from MCI 0.0 (data not shown). BMI was significant in MCI 1.5 (p = 0.02), and MCI 0.0 (p <0.001), and higher BMI increased the relative probability of classification into MCI 5.6.

**Table 5:** Parameter estimates for risk factors associated with each trajectory group in normal participants.

Trajectory group	Parameter	Estimate (SE)^a^	p-value
Norm 3.3	Intercept	0.93 (2.95)	0.75
	Age	0.06 (0.04)	0.16
	BMI	-0.12 (0.06)	0.06
	Sex	-0.41 (0.60)	0.49
	Education	-0.10 (0.11)	0.40
Norm 6.2	Intercept	-1.31 (3.60)	0.72
	Age	0.09 (0.04)	0.03
	BMI	0.01 (0.05)	0.90
	Sex	-0.98 (0.55)	0.08
	Education	-0.24 (0.11)	0.02
Norm 6.9	Intercept	8.10 (2.52)	0.001
	Age	-0.02 (0.04)	0.67
	BMI	-0.05 (0.06)	0.36
	Sex	-0.26 (0.59)	0.65
	Education	-0.29 (0.11)	0.01
Norm 9.1	Intercept	0.19 (3.34)	0.96
	Age	0.04 (0.05)	0.42
	BMI	-0.05 (0.06)	0.42
	Sex	-0.07 (0.62)	0.91
	Education	-0.10 (0.13)	0.44
Norm 12.9	Intercept	-2.88 (5.00)	0.57
	Age	-0.06 (0.06)	0.33
	BMI	0.02 (0.06)	0.69
	Sex	1.74 (0.73)	0.02
	Education	0.22 (0.15)	0.14

Note: all results of parameter estimates were derived by using Norm 9.4 as reference group for normal participants;

^a^ SE = standard error.

**Table 6:** Parameter estimates for risk factors associated with each trajectory group in MCI participants.

Trajectory group	Parameter	Estimate (SE)^a^	p-value
MCI 1.5	Intercept	2.70 (1.90)	0.16
	Apoe4	1.05 (0.33)	0.002
	Age	0.02 (0.02)	0.44
	BMI	-0.09 (0.04)	0.02
	Education	-0.08 (0.05)	0.14
MCI 0.0	Intercept	7.76 (1.91)	<0.001
	Apoe4	1.77 (0.47)	<0.001
	Age	-0.03 (0.02)	0.26
	BMI	-0.21 (0.05)	<0.001
	Education	-0.11 (0.07)	0.09
MCI 3.3	Intercept	-1.96 (2.97)	0.51
	Apoe4	0.40 (0.43)	0.35
	Age	0.06 (0.03)	0.07
	BMI	-0.01 (0.05)	0.83
	Education	-0.14 (0.07)	0.04
MCI 10.1	Intercept	-1.63 (3.52)	0.64
	Apoe4	-0.15 (0.56)	0.79
	Age	-0.01 (0.03)	0.75
	BMI	-0.03 (0.07)	0.64
	Education	0.13 (0.11)	0.22

Note: all results of parameter estimates were derived by using MCI 5.6 as reference group in MCI participants;

^a^ SE = standard error.

## Discussion

In this study, 6 latent trajectories with three main change patterns—stable, linear decline, and curvilinear decline—were identified for baseline normal participants, while five latent trajectories were found for baseline MCI. These results demonstrate that within same clinical diagnosis, distinct subgroups exist and may follow different developmental trajectories and experience disparate outcomes. The baseline scores that defined the trajectory groups were not a strong predictor of future cognitive status. Comparisons between cognitive status at enrollment and the end of follow-up by trajectories verified the prognosis of these potential trajectories, which emphasize the need for longitudinal data in making predictions about future cognition.

Consistent with the findings on memory change trajectories in participants from the Australian Imaging, Biomarkers, and Lifestyle (AIBL) study [27] and in Washington Heights Inwood Columbia Aging Project (WHICAP) [25], we identified stable and decline groups for baseline normal participants. Furthermore, our study also identified two curvilinear groups (Norm 9.1 and Norm 9.4), which accounts for about 28% of subjects in our sample. The Norm 9.1 group was stable during early follow-up, then showed a rapid decline in the following years. Several papers [28–30] described the sharp decline phenomenon, which suggested that some of those participants were initially cognitively stable but may have experienced a significant decline associated with cognitive impairment and dementia, and patients with rapid cognitive decline usually have a worse prognosis [29–31]. Compared to 65.5% and 50% participants assigned into stable groups for AIBL and WHICAP, respectively, we had proportionally fewer participants assigned to the stable groups (Norm 6.9 and Norm 12.9; about 30%). This inconsistency may be due to the larger number of trajectories identified in our study, the longer follow-up time in our analyses (9 years vs. 4.5 years in AIBL study and six years in WHICAP), as well as different inclusion and exclusion criteria within each study.

To our knowledge, this is one of a few studies to explore memory trajectories in MCI participants by using GBTM. Although most of the potential trajectory groups show a tendency to decline, 11% and 22% of participants in MCI 5.6 and MCI 10.1, respectively, were re-diagnosed with normal cognition at the end of follow-up, and 19% and 13% progressed to dementia, respectively, which reflects the heterogeneous outcomes often reported in MCI participants [32]. Consistent with other studies [4, 6, 32], our study supports that MCI may not be just the intermediate stage between normal cognition and dementia. The trajectories in MCI 1.5 and MCI 0.0 began with low scores, and the majority (73% in MCI 1.5 and 71% in MCI 0.0) progressed to dementia, which may indicate the participants in these two groups were already at a late stage of MCI at enrollment. Based on our results, the rate of incident dementia from MCI may be correlated with the baseline mean of RAVLT 30-minute delayed recall. The higher the baseline mean value, the lower the incidence rate. Overall, the 9-year cumulative incidence of dementia from MCI was 53% (roughly 8% per year). The annual rate is comparable to the rate for the 5-year cumulative incidence of dementia from MCI reported in specialist centers (39%, or roughly 9% per year) [4].

Different demographic variables were associated with trajectory membership for normal and MCI participants. For normal baseline participants, older age and less education were significantly associated with being in the “linear decline” group (Norm 3.3), and participants with less education were relatively more likely to be in Norm 6.9. Being female was associated with a stable trajectory (Norm 12.9), which is shown in Table 6 (female 77%), but was inconsistent with Lin’s study [33]. In baseline MCI participants, genetic risk factor *APOE*-ɛ4 allele and/or lower BMI was associated with lower memory scores (MCI 1.5 and MCI 0.0).

### Strengths and limitations

The strengths of this study included relatively large baseline sample size (219 for normal and 372 for MCI), frequent clinical assessments, standardized diagnostic criteria for cognitive status, and standardized data collection procedure across multiple study sites. This allowed a rigorous investigation of memory trajectories and their relationship with risk or protective factors using long follow-up and multiple visits (up to 12 visits for over nine years).

One limitation of the study sample is that the participants in ADNI may not be representative of the general population of older adults in the United States. We focused only on participants from ADNI1 to obtain participants with longer follow-up, so we excluded the early MCI participants recruited in ADNIGO, and late MCI participants enrolled in ADNI2 due to insufficient follow-up. The diagnosis of MCI was made without further specifying the subtype of MCI (i.e., amnestic, nonamnestic, single domain, multiple domains). Thus, a more homogeneous set of trajectories may exist within subtypes of MCI participants. Furthermore, the specific trajectory groups defined in our analysis are not likely to generalize to other populations. In the future studies, we aim to validate these trajectories using MRI or biomarker data and identify trajectories for subsets of MCI participants (i.e., early mild cognitive impairment (EMCI), late mild cognitive impairment (LMCI)).

Another limitation is the uncertainty of group membership. Even though the average posterior probability is high, the uncertainty of group assignment may lead to bias [5, 34]. Also in general, although demographics and baseline scores may provide some guidance, patients cannot be assigned with accuracy to any trajectory at an initial visit but rather only after the subject has been followed for several assessments.

## Conclusion

Group based trajectory modeling can be used to identify latent subgroups of participants based on memory trajectory. The relationship between trajectory group and cognitive status at end of follow-up confirmed that memory trajectory is an excellent indicator of dementia risk. If trajectory group membership can be identified reliably during early follow-up, such work will allow clinicians to monitor or predict progression of individual patient’s cognition. This work also shows the importance of longitudinal cognitive testing and monitoring.

## References

[1] Gauthier S., et al, Mild cognitive impairment. Lancet, 2006 367(9518): p. 1262–70. 10.1016/S0140-6736(06)68542-5 16631882

[2] Abner E.L., et al, Outcomes after diagnosis of mild cognitive impairment in a large autopsy series. Ann Neurol, 2017 81(4): p. 549–559. 10.1002/ana.24903 28224671 PMC5401633

[3] Wolf H., et al, The prognosis of mild cognitive impairment in the elderly. J Neural Transm Suppl, 1998 54: p. 31–50. 9850913 10.1007/978-3-7091-7508-8_4

[4] Mitchell A.J. and Shiri-Feshki M., Rate of progression of mild cognitive impairment to dementia—meta-analysis of 41 robust inception cohort studies. Acta Psychiatr Scand, 2009 119(4): p. 252–65. 10.1111/j.1600-0447.2008.01326.x 19236314

[5] Jones BL, N. D., Roeder K A SAS precedure based on mixture models for estimating developmental trajectories. Soc Methods Res, 2001 29.

[6] Xie H., Mayo N., and Koski L., Identifying and characterizing trajectories of cognitive change in older persons with mild cognitive impairment. Dement Geriatr Cogn Disord, 2011 31(2): p. 165–72. 10.1159/000323568 21346357

[7] DS N., *Group-Based Modeling of Development*. 2005: Harvard University Press.

[8] Nagin D.S. and Odgers C.L., Group-based trajectory modeling in clinical research. Annu Rev Clin Psychol, 2010 6: p. 109–38. 10.1146/annurev.clinpsy.121208.131413 20192788

[9] Roeder B.L.J.D.S.N.K., A SAS Procedure Based on Mixture Models for Estimating Developmental Trajectories. Sociological Methods & Research 2001 29(3): p. 10.

[10] McKhann G., et al, Clinical diagnosis of Alzheimer’s disease: report of the NINCDS-ADRDA Work Group under the auspices of Department of Health and Human Services Task Force on Alzheimer’s Disease. Neurology, 1984 34(7): p. 939–44. 6610841 10.1212/wnl.34.7.939

[11] Levin A.P.A.S.J., The Rey Auditory Verbal Learning Test: normative data for the Arabic-speaking population and analysis of the differential influence of demographic variables. Psychology & Neuroscience, 2012 5.

[12] *Encyclopedia of Clinical Neuropsychology*, Kreutzer J., DeLuca John, Editor. p. 2174–2175.

[13] Klekociuk S.Z. and Summers M.J., The learning profile of persistent mild cognitive impairment (MCI): a potential diagnostic marker of persistent amnestic MCI. Eur J Neurol, 2014 21(3): p. 470–7, e23–4 10.1111/ene.12333 24372923

[14] Lezak M.D., Howiesen D. B. & Loring D. W., *Neuropsycholigical Assessment* *(*4th edition*)* 2004.

[15] Hamdan S.S.M.a.A.C., The Rey Auditory Verbal Learning Test: normative data for the Brazilian population and analysis of the influence of demographic variables. PSYCHOLOGY & NEUROSCIENCE, 2010 3(1): p. 7.

[16] Messinis L., et al, Age and education adjusted normative data and discriminative validity for Rey’s Auditory Verbal Learning Test in the elderly Greek population. J Clin Exp Neuropsychol, 2016 38(1): p. 23–39. 10.1080/13803395.2015.1085496 26588427

[17] Chang Y.L., et al, Brain substrates of learning and retention in mild cognitive impairment diagnosis and progression to Alzheimer’s disease. Neuropsychologia, 2010 48(5): p. 1237–47. 10.1016/j.neuropsychologia.2009.12.024 20034503 PMC2851550

[18] Zhao Q., et al, Auditory Verbal Learning Test is Superior to Rey-Osterrieth Complex Figure Memory for Predicting Mild Cognitive Impairment to Alzheimer’s Disease. Curr Alzheimer Res, 2015 12(6): p. 520–6. 26027810 10.2174/1567205012666150530202729

[19] Tierney M.C., Moineddin R., and McDowell I., Prediction of all-cause dementia using neuropsychological tests within 10 and 5 years of diagnosis in a community-based sample. J Alzheimers Dis, 2010 22(4): p. 1231–40. 10.3233/JAD-2010-100516 20930315

[20] Eckerstrom C., et al, A combination of neuropsychological, neuroimaging, and cerebrospinal fluid markers predicts conversion from mild cognitive impairment to dementia. J Alzheimers Dis, 2013 36(3): p. 421–31. 10.3233/JAD-122440 23635408

[21] Ferman T.J., et al, Neuropsychological differentiation of dementia with Lewy bodies from normal aging and Alzheimer’s disease. Clin Neuropsychol, 2006 20(4): p. 623–36. 10.1080/13854040500376831 16980250

[22] Weiner M.W., et al, The Alzheimer’s disease neuroimaging initiative: progress report and future plans. Alzheimers Dement, 2010 6(3): p. 202–11.e7. 10.1016/j.jalz.2010.03.007 20451868 PMC2927112

[23] Schmidt, M., Rey Auditory Verbal Learning Test (RAVLT). 1996.

[24] Barzotti T., et al, Correlation between cognitive impairment and the Rey auditory-verbal learning test in a population with Alzheimer disease. Arch Gerontol Geriatr Suppl, 2004(9): p. 57–62. 10.1016/j.archger.2004.04.010 15207397

[25] Zahodne L.B., et al, Dementia Risk and Protective Factors Differ in the Context of Memory Trajectory Groups. J Alzheimers Dis, 2016 52(3): p. 1013–20. 10.3233/JAD-151114 27079709 PMC4884159

[26] Nagin, D.S., Group-Based Modeling of Development. 2005.

[27] Pietrzak R.H., et al, Trajectories of memory decline in preclinical Alzheimer’s disease: results from the Australian Imaging, Biomarkers and Lifestyle Flagship Study of ageing. Neurobiol Aging, 2015 36(3): p. 1231–8. 10.1016/j.neurobiolaging.2014.12.015 25585532

[28] Hui J.S., et al, Rate of cognitive decline and mortality in Alzheimer’s disease. Neurology, 2003 61(10): p. 1356–61. 14638955 10.1212/01.wnl.0000094327.68399.59

[29] Carcaillon L., et al, Fast cognitive decline at the time of dementia diagnosis: a major prognostic factor for survival in the community. Dement Geriatr Cogn Disord, 2007 23(6): p. 439–45. 10.1159/000102017 17476100

[30] SotoM.E., et al, Rapid cognitive decline in Alzheimer’s disease. *Consensus paper*. J Nutr Health Aging, 2008 12(10): p. 703–13. 1904364510.1007/BF03028618

[31] Gauthier S., et al, Aggressive course of disease in dementia. Alzheimers Dement, 2006 2(3): p. 210–7. 10.1016/j.jalz.2006.03.002 19595889

[32] Palmer K., Fratiglioni L., and Winblad B., What is mild cognitive impairment? Variations in definitions and evolution of nondemented persons with cognitive impairment. Acta Neurol Scand Suppl, 2003 179: p. 14–20. 12603245 10.1034/j.1600-0404.107.s179.2.x

[33] Lin K.A., et al, Marked gender differences in progression of mild cognitive impairment over 8 years. Alzheimers Dement (N Y), 2015 1(2): p. 103–110. 26451386 10.1016/j.trci.2015.07.001 PMC4593067

[34] Roeder Kathryn L. K.G., and Nagin Daniel S., Modeling Uncertainty in Latent Class Membership: A Case Study in Criminology. Journal of the American Statistical Association, 1999 94: p. 11.

